# Quantification of the Organic Acids in Hawthorn Wine: A Comparison of Two HPLC Methods

**DOI:** 10.3390/molecules24112150

**Published:** 2019-06-07

**Authors:** Yingying Han, Jinhua Du, Jie Li, Miaomiao Li

**Affiliations:** College of Food Science and Engineering, Shandong Agricultural University, Tai’an 271018, China; hanyingying9203@163.com (Y.H.); lijie_amy@163.com (J.L.); limiaomiao0538@163.com (M.L.)

**Keywords:** hawthorn wine, organic acids, high-performance liquid chromatography methods, enzymatic method

## Abstract

Hawthorn wine is rich in anthocyanins, polyphenols, flavonoids and other macromolecular substances, which results in difficulty to rapidly determine organic acids in the wine. An enzymatic method is accurate but expensive and not able to quantify all of the organic acids simultaneously. Therefore, in this study, two HPLC methods were applied to quantify the organic acids in the wine with the enzymatic method as a reference. Seven organic acids were found with the enzymatic method including citric, succinic, l-malic, acetic, lactic, pyruvic, and fumaric acids, in which citric and succinic acid accounted for more than 80% of the total acids. By an 87H column equipped with DAD (diode array) detector at 215 nm (HPLC method 1), only citric and lactic acids were quantified accurately and the elution period was shortened from 100 min to 20 min by removing the impurity in the sample with a LC-18 SPE(solid-phase extraction) tube. While citric, succinic, l-malic, acetic, pyruvic, and fumaric acids were quantified reliably by a dC18 column equipped with DAD detector at 210 nm (HPLC method 2), with the sample requires only dilution and filtration before injection. It was suggested that HPLC method 2 was an effective method to quantify the organic acids in hawthorn wine. The method provides a choice for accurate quantification of organic acids in hawthorn wine or other drinks, and would be helpful for controlling the quality of hawthorn wine.

## 1. Introduction

Hawthorn mostly grows around the temperate region of the world. Its fruits play an important role in promoting digestion [1], reducing blood tension [2], resisting oxidation [3], preventing cardiovascular diseases [4,5] and type II diabetes [6]. Although hawthorn has many functions for human health, strong acid-sensing makes it not widely suitable to be eaten raw. The total acidity of fresh hawthorn fruits is as much as 11.8 g/100 g dry weight [7]. To meet the challenge, the hawthorn is widely used to ferment wine. Nowadays, hawthorn wine is very popular in China for not only its natural nutrients but also its unique flavor. Additionally, the profile and concentration of organic acids are important parameters in relation to the characteristics and flavor of hawthorn wine owing to their unique flavor and influence on taste balance, chemical and microbial stability [8]. As is known, appropriate amount of organic acids can give hawthorn wine a fresh taste and appropriate sweet-sour balance; while a tart taste will appear if they are excessive. Therefore, quantification of organic acids plays an important role in controlling the quality of hawthorn wine. Up to now, the studies of hawthorn wine have been mainly focused on screening the optimal yeast for fermentation [9], analyzing the content of phenolic substances and their antioxidant properties during storage or fermentation using different yeasts [10,11,12] and the methods to reduce methanol content [13]. However, the analysis of organic acids in hawthorn wine was rarely reported.

The main methods reported to evaluate organic acids in fruits, juice and wine are the enzymatic method [14,15,16], capillary electrophoresis [17,18], gas chromatography [19], liquid chromatography [20] and ion chromatography [21]. The enzymatic method is highly specific and many commercial kits are available for determining certain organic acids, such as citric acid, succinic acid, l-malic acid, acetic acid, lactic acid, pyruvic acid and fumaric acid. However, these kits are expensive, time consuming and cannot be used to quantify all of the organic acids in the samples simultaneously. Better separation can be obtained with ion chromatography and gas chromatography, but they were hard to use widely because of the high consumption, harsh operating conditions, as well as the tedious pretreatment. Capillary electrophoresis presents not only advantages including high resolution, simplicity and short analysis times, but also disadvantages, such as lower reproducibility. High performance liquid chromatography (HPLC) is the most popular method for analyzing organic acids because of the simplicity, speed and stability. The C18 column, as a common HPLC column, was used for separating organic acids frequently by phosphate solution as mobile phase [22,23]. Additionally, ion exchange column such as HPX-87H has been used to separate organic acids because it can separate sugars and acids simultaneously [20,24].

Based on our previous research, hawthorn wine is rich in anthocyanins, polyphenols and flavonoids [10] and other macromolecular substances such as pectin [13], which results in problems to rapidly determine organic acids in the wine. Organic acids separated with HPLC are usually detected with ultraviolet detector (UV) at wavelengths below 220 nm where the carboxyl groups have an absorption band [25]. However, coexisting substances can interfere with organic acids in the low wavelength range especially those with conjugate double bonds, such as phenols which still have strong UV absorption even at low concentrations. Therefore, ten hawthorn wines were fermented by wine yeast. Two HPLC methods were compared to quantify organic acids in the wines, with enzymatic method working as a reference method. By the enzymatic method seven organic acids was quantified including citric, succinic, l-malic, acetic, lactic, pyruvic, and fumaric acids, respectively. Two HPLC methods applied were an 87H column equipped with a DAD detector at 215 nm (method 1) and a dC18 column equipped with a DAD detector at 210 nm (method 2). This study is aiming to quantify organic acids in hawthorn wine accurately, rapidly and simultaneously, and control the quality of hawthorn wine by monitoring the organic acids. The varietal characteristics, processing conditions, microbial contamination of hawthorn wine and ripeness levels of the hawthorn fruits can be understood by monitoring the organic acids content.

## 2. Results and Discussion

### 2.1. Physicochemical Analysis of Hawthorn Wine

The physicochemical indexes of the ten hawthorn wines were shown in Table 1. The alcohol content varied from 14.69% to 15.45% by volume; residual sugar content was 3.51–5.75 g/L; sugar-free extract was 32.35–37.45 g/L, and pH value ranged from 3.08 to 3.28. It was found that the total acidity was relatively high and range from 13.15 g/L to 15.16 g/L. Appropriate acidity gives the wine a fresh taste and benefits the microbial stability of the wine while excessive acidity will give the wine a tart taste. Therefore, accurate and rapid quantification of organic acids plays an important role in maintaining a well balance of sugar and acid and controlling the quality of hawthorn wine.

### 2.2. Identification of Organic Acids in Hawthorn Wine with the Enzymatic Method

As shown in Table 2, citric acid (7967.6–9821.3 mg/L) was the most abundant organic acid in hawthorn wine, accounting for more than 70% of the total organic acid. The followed one is succinic acid (1327.7–1694.9 mg/L), which accounted for about 10% of the total organic acid. Hawthorn wine was also rich in l-malic acid (603.2–983.2 mg/L) and acetic acid (523.5–818.8 mg/L), which accounted for about 6% and 5%, respectively. In hawthorn wines, the content of lactic acid (72.7–135.4 mg/L) was less than 1% of the total acid. Additionally, only a trace amount of pyruvic acid (4.8–7.4 mg/L) and fumaric acid (2.5–3.7 mg/L) was detected. However, in the hawthorn fruits (Table 5), citric acid (26.95 mg/g) was the most abundant organic acid, accounting for 85% of the total organic acid, with the followed one being l-malic acid (3.29 mg/g).

Succinic acid, acetic acid, lactic acid and pyruvic were also detected in hawthorn wine, which accounting for 1.99%, 2.36%, 0.19% and 0.19%, respectively. Comparing organic acids in hawthorn fruits and hawthorn wines, citric acid and l-malic acid contents decreased during fermentation; while succinic acid increased significantly, as well as acetic acid and lactic acid. The citric acid and l-malic acid were the main acids in hawthorn fruits. Previous study indicated that the content of citric acid and l-malic acid in hawthorn fruits reached up to 85% of the total acidity [7]. The succinic acid, acetic acid and lactic acid not only came from the raw material but also produced by the yeast during the alcoholic fermentation. Fumaric acid was an indicator of microbial contamination in clear apple juice concentrate [26,27]. Only trace amount of fumaric acid was detected in both hawthorn wine fruits and hawthorn wines. Pyruvic acid was an important intermediate between EMP (Embden–Meyerhof-Parnas) pathway and TCA cycle (tricarboxylic acid cycle) as well as other metabolisms; it was converted to organic acid and other substances during the fermentation. Although the content of these organic acids analyzed by enzymatic method was reliable, these kits were expensive, and the method is time consuming and cannot quantify all of the organic acids simultaneously. It is commonly used as a reference method in food analysis [28]. In contrast, the HPLC method was widely applied to quantify organic acids in food. However, the detection of organic acids in hawthorn wine by HPLC has been rarely reported. Therefore, two popular HPLC methods were applied to quantify organic acids following.

### 2.3. Validation of the two HPLC Methods

To validate the two HPLC methods, proper quality assurance/quality control (QA/QC) procedures were applied. As shown in Table 3, a good linear range of the two HPLC methods were showed where the values for the correlation coefficient (R^2^) were 0.9995–1.0000 and their concentration range encompasses the expected analyte concentrations in the measured samples. According to Konieczka [29], the R^2^ values for the calibration curves must be greater than 0.999, which verifies that the linearity obtained in this study for the response to external standards was adequate for the intended purpose. The precision parameter of the HPLC method 1 showed a coefficient of variation ranged from 0.76 to 4.21 for hawthorn wine. And the precision parameter of the HPLC method 2 ranged from 0.26 to 1.73. The variation coefficient of pyruvate was relative large with HPLC method 1 but still lower than the maximum limit of 5% [30]. The HPLC method 2 was better than method 1 in precision for its good reproducibility. Recovery was obtained from the results of six injections of 5.0 mL of the hawthorn wine samples mixture with 5.0 mL of a mix organic acids standards solution. The recovery was calculated comparing the result obtained analytically for each compound with the initial concentration in the spiked sample. The recovery percentages (R%) values for the wine ranged from 83.6 to 101.2 analyzed with the HPLC method 1, and ranged from 98.2 to 108.5 analyzed with the HPLC method 2. The recovery rate of acetic acid (83.6) was low when analyzed with HPLC method 1; it is speculated that there might be material interference. The LOD (limit of detection) and LOQ (limit of quantification) were used to demonstrate the ability of a method to detect and quantify low concentrations of a substance, respectively. The LOD and LOQ values were considered suitable for the use of this methodology in quantification of organic acids in hawthorn wine.

### 2.4. Analysis of Organic Acids with HPLC Method 1

**Figure 1:**
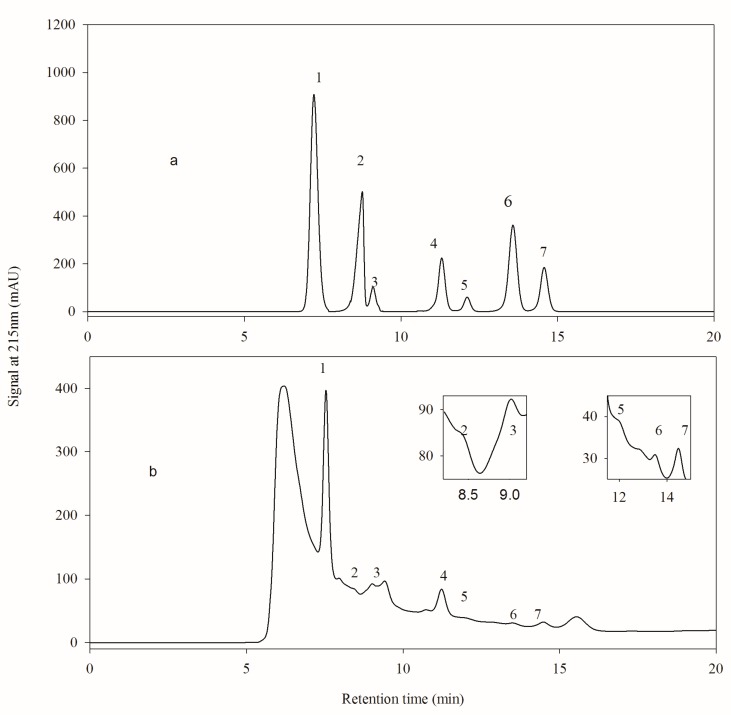
Analysis of organic acids with HPLC method 1. (**a**) Chromatogram of organic acid standards; (**b**) Chromatogram of hawthorn wine. Peak 1, 2, 3, 4, 5, 6 and 7 were citric acid, pyruvic acid, l-malic acid, succinic acid, lactic acid, fumaric acid, and acetic acid.

As shown in Figure 1a, seven organic acid standards, including citric acid, pyruvic acid, l-malic acid, succinic acid, lactic acid, fumaric acid and acetic acid, were well separated with HPLC method 1. However, the chromatogram of one hawthorn wine sample exhibited an unstable baseline and reduced separation because of the presence of interfering materials. In the study of Coelho et al. [24], an unknown substance eluted followed by l-malic acid led to a weak separation and it was difficult to increase the separation degree of l-malic acid by adjusting the mobile phase. Combined Table 3 with Figure 1b, the content between individual organic acid in hawthorn wine varied greatly. The citric acid content was much higher than the others, leading to the instability and poor separation of pyruvic acid, l-malic acid and fumaric acid, which could not be improved even by adjusting the acids concentration in the samples. The content of fumaric acid and pyruvic acid was poor in hawthorn wines, and the baseline drift will lead to the increase of error. 

### 2.5. Analysis of Organic Acids with HPLC Method 2 

**Figure 2:**
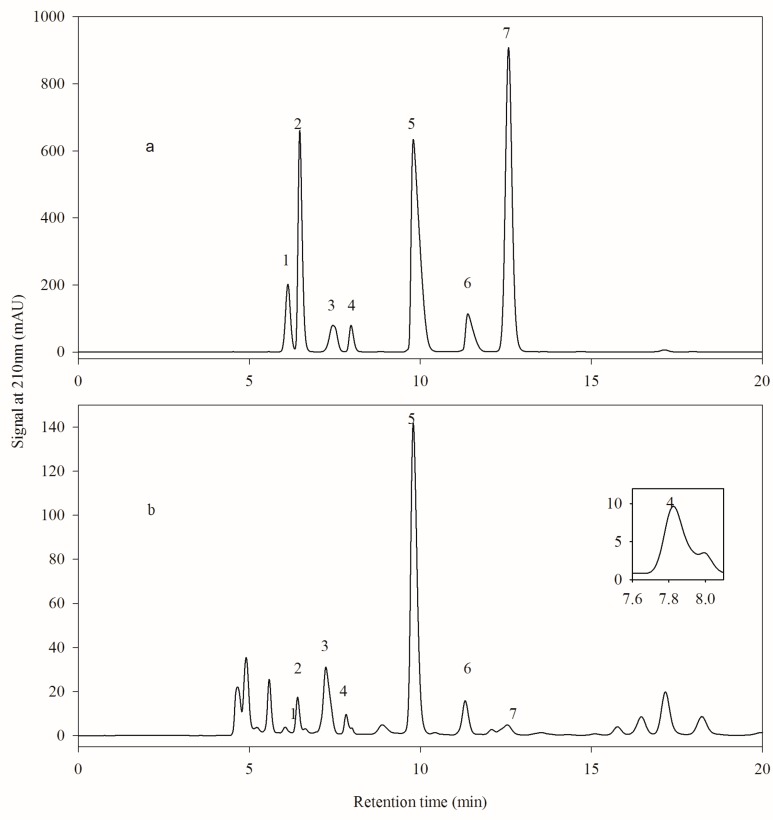
Analysis of organic acids with HPLC method 2. (**a**) Chromatogram of organic acid standards; (**b**) Chromatogram of the hawthorn wine. Peak 1, 2, 3, 4, 5, 6 and 7 were pyruvic acid, l-malic acid, lactic acid, acetic acid, citric acid, succinic acid, and fumaric acid.

As indicated in Figure 2a, the seven organic acid standards mentioned in 2.4 were well separated with HPLC method 2. By analyzing the same hawthorn wine sample used in Figure 1, stable baseline and effective separation except acetic acid were realized as showed in Figure 2b. An unknown peak at retention time of 8.0 min in Figure 2b appeared behind the peak of acetic acid, leading to a weak separation. Although the elution period of organic acid standards is only 13 min, many weak polar materials were eluted followed by the last peak of fumaric acid, resulting in up to 100 min analysis period for one sample as exhibited in Figure 4b.

The difference in separation principle between these two HPLC methods led to significant differences in sample separation. For HPLC method 1, the main problems were unstable baseline, poor separation in pyruvic acid, l-malic acid and fumaric acid and the long elution period. For HPLC method 2, the problems were the long elution period and poor separation degree in acetic acid. The main reason for these problems mentioned above is due to the poor selectivity of diode array detector at the low wavelength range [25]. Phenols, nucleotide phosphates and the substances containing conjugated double bond display intensive absorption in this ultraviolet range. Thus, they are able to disturb the determination even at a very low level. Another reason might be that hawthorn wine is a complex system containing vitamins, polyphenols, pigments, polysaccharides [11] and other interfering substances. Therefore, LC-18 SPE tube pretreatment would be applied in the following pretreatment.

### 2.6. Pretreatment of the Samples with LC-18 SPE Tube

#### 2.6.1. Recovery of Organic Acids Treated with LC-18 SPE Tube

As indicated in Table 4, precision of the organic acids in hawthorn wine was reduced after purification pretreatment, especially with HPLC method 1. It was speculated that the impurity removed effectively by the purification pretreatment and the filtration speed will affect the degree of purification when analyzed with HPLC method 1. There were no large changes in the LOD and the LOQ because the signal-to-noise ratio did not change so much. The recovery rate of organic acid standards analyzed with HPLC method 1 was 90.8–101.6%, demonstrating that the LC-18 SPE tube will not result in organic acids loss. However, the recovery of some organic acids in the samples was out of the reasonable range. The recovery rate of pyruvate acid was 139.9% with a relatively high deviation (34.9%). It is speculated that the main reason would be the flat baseline and the increased separation after purification. On the other hand, the tiny elution peak of pyruvate acid led to the large deviation owing to the lower content in these samples. The recovery rate of lactic acid in the sample was only 71.4%, while the standard was 101.6%. It is considered that the low recovery rate is due to the unknown substances overlapped on the peak of lactic acid and the impurities can be removed after purification. Similar to lactic acid, acetic acid also has the problem. 

The recovery of the standards analyzed with method 2 was 91.2–103.1%. Similarly, high sample recovery (94.7–101.7%) suggested that the use of LC-18 SPE tube has little impact on the sample. Organic acids can be accurately quantified for a high-fortified recovery (98.2–108.5%). 

#### 2.6.2. Effect of LC-18 SPE Tube Pretreatment on the Analysis of HPLC Method 1

**Figure 3:**
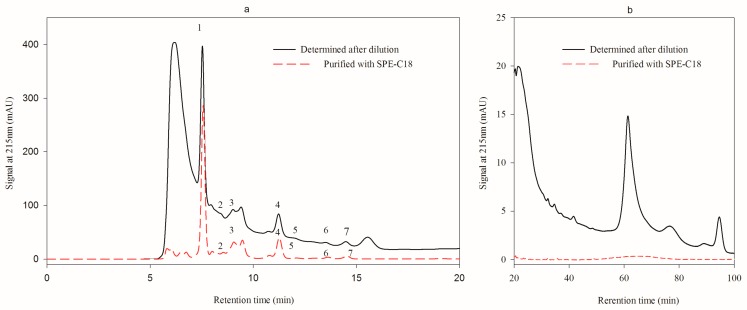
Effect of LC-18 SPE tube pretreatment on the analysis of HPLC method 1 (**a**), chromatogram of the first 20 min elution, Peak 1, 2, 3, 4, 5, 6 and 7 were citric acid, pyruvic acid, l-malic acid, succinic acid, lactic acid, fumaric acid, and acetic acid; (**b**), chromatogram of the 20–100 min elution.

The sample exhibited a quite clean chromatogram after being pretreated with LC-18 SPE tube (Figure 3a,b), indicating the impurities were efficiently removed. As a result, baseline becomes stable, the separation degree of fumaric acid and pyruvic acid was increased to the same extent and the analysis period was reduced from 100 min to 20 min (Figure 3b). However, the unknown substance eluted followed by l-malic acid could not be removed. 

#### 2.6.3. Effect of LC-18 SPE Tube Pretreatment on the Analysis of HPLC Method 2

**Figure 4:**
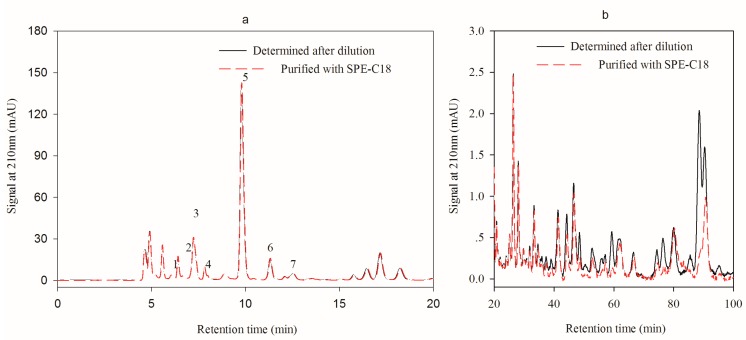
Effect of LC-18 SPE tube pretreatment on the analysis of HPLC method 2 (**a**), chromatogram of the first 20 min elution, Peak 1, 2, 3, 4, 5, 6 and 7 were pyruvic acid, l-malic, lactic acid, acetic acid, citric acid, succinic acid and fumaric acid; (**b**), chromatogram of the 20–100 min elution.

As indicated in Figure 4a,b, the LC-18 SPE tube has little impact on the analysis period. It was probably because of the same filling material and the similar adsorption capacity of the tube and the dC18 column. However, LC-18 SPE tube has the advantage that the compounds binding irreversibly to the tube can be efficiently removed and thus contamination of the analytical column is minimized. Although the addition of organic solvent such as methanol to the mobile phase can shorten the analysis time when separating with C18 column, it needs a larger proportion of organic solvent. This operation leads to the significantly increased column pressure owing to the low solubility of salt in organic phase. It was reported that organic acids bound to the NH_2_ solid phase extraction column could effectively wipe off the interference and shorten the sample analysis time when being eluted with phosphoric acid [31]. However, solid phase extraction treatment is time-consuming and need a large amount of organic reagents.

### 2.7. Quantification of Arganic Acids with Two HPLC Methods

As demonstrated in Table 2, the citric acid content quantified with HPLC method 2 showed no significant difference (*p* > 0.05) from that with HPLC method 1 and the enzymatic method. However, the content of succinic acid in the same hawthorn wine sample was significantly different between two HPLC methods. Taking the enzymatic method as a reference, the quantitative result of HPLC method 2 was more credible. As for l-malic acid, there was no significantly different between two HPLC methods and enzymatic method in most samples, Lower separation was obtained by HPLC method 1, it might be due to the similar peak height and peak area of the interfering substances with that of l-malic acid. Furthermore, the acetic acid content by HPLC method 1 was relatively higher than that by HPLC method 2 and enzymatic method. It was speculated that substances unknown were eluted with the acetic acid at the same time, which was in consistent with the low fortified recovery of acetic acid (Table 4). The situation of lactic acid was similar to that of succinic acid, effective separation and reasonable fortified recovery were obtained by HPLC method 2, but the content was much higher than those obtained with HPLC method 1 and the enzymatic method. Briefly, HPLC method 1 quantified citric acid and lactic acid credibly; while HPLC method 2 quantified the main organic acids in hawthorn wine credibly, including the citric acid, succinic acid, l-malic acid, acetic acid, fumaric acid, and pyruvic acid. 

The HPLC method 2 was a better method not only for the accuracy in quantifying the main organic acids in hawthorn wine but also for its environmental friendliness according to Green Analytical Procedure Index [32] and the Analytical Eco-Scale [33]. The mobile phase of the HPLC method 2 was phosphate and water, and the sample pretreatment just need dilution and filtration. No organic reagents were used throughout the whole preparation procedure, which would not damage the environment. Solid phase extraction with C18 columns had been used in order to minimise the contamination in column [34]. This was an excellent method for removal of lipophilic compounds like pigments, which bind strongly to the SPE column. NH_2_ solid phase extraction columns were another choice for purifying samples. However, in contrast to C18 columns, the organic acids were quantitatively retained on the NH_2_ columns and needed to be eluted with high levels organic solvents subsequently [34,35,36]. Therefore, this was an accurate and environmental friendly approach to quantify organic acids in hawthorn wine.

## 3. Materials and Methods

### 3.1. Materials and Reagents

Hawthorn fruits used to brew the wine were purchased from the local fruit market (Taian, China), organic acids contents of them were shown in Table 5. Commercial wine yeast SIHA Active Yeast 3 (Saccharomyces cerevisiae WET 136) reputed to hawthorn wine making was purchased from E. Begerow GmbH & Co (Langenlonsheim, Germany). Standards including citric acid (99.5%), lactic acid (92%), acetic acid (99.7%), fumaric acid (99%) and succinic acid (99%) were purchased from Sigma Aldrich (St. Louis, MO, USA). l-malic acid standard (98%) was obtained from Aladdin (Shanghai, China). Pyruvic acid (98%) was ordered at Yuanye (Shanghai, China). Organic acid assay kits were purchased from Megazyme (Bray, Ireleand), including acetic acid (ACS Manual Format, K-ACET; accepted by ISO, EN, CUMSA, IFU, MEBAK), citric acid (K-CITR; accepted by AOAC, OIV, EU, ISO2963, IFU22, MEBAK), succinic acid (K-SUCC; accepted by EEC), d-/l-lactic acid (K-DLATE; accepted by ISO, EEC, DIN, GOST, IDF, EN, OIV, AIJN, IFU, MEBAK) and l-malic acid (Manual Format, K-LMAL; accepted by AOAC, EEC, EN, NF, NEN, DIN, GOST, OIV, AIJN, IFU, MEBAK) assay kits. Plant pyruvate ELISA and fumaric ELISA acid kits were purchased at Jiangsu Jingmei Biological Technology Co. Ltd. (Jiangsu, China).

### 3.2. Technological Process of Hawthorn Wine Making

The making of hawthorn wine was performed according to He et al. [10] and with some modification. Well-matured hawthorn fruits were selected, which was characterized as having a dark red fruit skin and a strong fruit aroma. The fruits were washed with tap water and 100 mg/L of KMnO_4_, followed by flushing with sterile water, draining and crushing by a crusher to 3–5 mm size particles. The crushed hawthorn fruit must was mixed with a 40% aqueous solution of white granulated sugar (mash) in a ratio of 1:1.2 in a fermenter with an 80% loading. Yeast SIHA Active Yeast 3 in a ratio of 30 g/100 kg by chaptalized must was added into the must to obtain a total inoculated population up to 5.0 × 10^6^ cells/mL. The fermentation temperature was conducted at 22–24 °C. The fermenting mash was gently stirred once a day. The pomace and seed were separated from the fermented juice when the residual sugar in the must had no longer decreased over three consecutive days. After aging, the hawthorn wine was clarified with 1.2 g/L bentonite. The wine was filtered and bottled. Ten hawthorn wines were made for organic acids analysis.

### 3.3. Chemical Analysis

Physicochemical indexes of hawthorn wine were determined including alcohol, reducing sugar, sugar-free extract, titratable acidity and pH value. The alcohol and sugar-free extract of the hawthorn wine were performed with the method of pycnometer according to the methods of the National Standards of the People’s Republic of China GB/T 15038. The automatic determine instrument of reducing sugar (SGD-IV-D, China) was used to quantify reducing sugar. The total acidity was performed by potentiometric titration, with pH 8.20 as titration end-point titrated with 0.1 M sodium hydroxide standard solution and calculated according to the volume of sodium hydroxide consumed. The pH value was measured directly in the wine by the laboratory recording pH meter (Mettler Toledo, Switzerland).

### 3.4. Identification of Organic Acids with the Enzymatic Method

Identification of organic acids in hawthorn wines with the enzymatic method was conducted according to the manufacturer’s instruction. A multi-mode microplate reader (SpectraMaxR M5, Molecular Devices, San Jose, CA, USA) was used for the measurements. The pretreatment and determination of samples were conducted according to the manufacturer’s manual.

### 3.5. Quantification of Organic Acids with HPLC Method 1

#### 3.5.1. Pretreatment of Samples

Samples were centrifuged at 5000 rpm for 10 min; the resulting supernatant was aliquoted into two parts after a five-fold dilution with purified water. Part one was sequentially filtered through a 0.45 μm and a 0.22 μm pore size membrane filters (Nylon PES, Tianjin, China) for further analysis. Part two was passed through a LC-18 SPE Tube (SupelcleanTMLC-18 SPE Tubes, Supelco, USA) activated by menthol in advance according to the manufacturer’s instruction and then sequentially filtered by a 0.45 μm and a 0.22 μm pore size membrane filters (Nylon PES, Tianjin, China) for further analysis.

#### 3.5.2. Chromatographic Condition 

Quantification of organic acids by Aminex HPX-87H column (300 × 7.8 mm, Bio-Rad, Hercules, CA, USA) was performed as described previously [34] with some modifications. The mobile phase was 3 mM sulfuric acid aqueous solution. Sample (20 µL) was separated on a column set at 42 °C with a flow rate of 0.6 mL/min; and the absorbance at 215 nm was monitored by an SPD-M20A detector (Shimadzu, Japan). The HPLC system consisted of two pumps (LC-20 AT, Shimadzu, Kyoto, Japan), a degasser (DGU-20A, Shimadzu), an autosampler (SIL-20 A, Shimadzu) and a column oven (CTO-20A, Shimadzu). Chromatograms were evaluated with the Clarity software package (LabSolutions, Shimadzu). This method was termed as HPLC method 1.

#### 3.5.3. Quantification of Organic Acids

The peaks of organic acids were identified by their retention time, and quantification was determined using an external standard curve. Six concentration points were used to plot the calibration curves. The limit of detection was the minimal concentration of the analyte giving a peak height with only three times of the noise base line, and 10 times for the limit of quantification. Linearity of organic acids signal is presented in Table 3.

### 3.6. Quantification of Organic Acids with HPLC Method 2

#### 3.6.1. Pretreatment of Samples

Samples were centrifuged at 5000 rpm for 10 min to harvest the supernatant which was thereafter divided into two parts after being diluted fourfold using purified water. The pretreatment of these two parts was as same as Section 3.5.1.

#### 3.6.2. Chromatographic Condition

Organic acids in the samples were separated on a dC18 column (250 × 4.6 mm, 5 µm, Waters, Atlantis, MA, USA) as described previously [23] with some modifications. The mobile phase was 0.02 M KH_2_PO_4_ (pH 2.8) with a flow rate of 0.7 mL/min and the column temperature was 35 °C. The injection volume was 10 μL. Samples were detected with DAD (Shimadzu, Japan) at a wavelength of 210 nm in the same HPLC system. This method was termed as HPLC method 2.

#### 3.6.3. Quantification of Organic Acids

Under the chromatographic conditions described above, calibration curves were determined for the mixture of organic acids standard solutions with five different concentrations. Table 3 shows the linear equation, the range of linearity and the determination coefficients of organic acids. 

### 3.7. Pretreatment with LC-18 SPE Tube

The effect of LC-18 SPE tube on organic acid standards was verified through the recovery experiment. The mixture of standards containing seven organic acids was diluted into different concentrations, and each concentration was divided into two groups. The pretreatment of these two groups was as same as Section 3.5.1. The calculation was performed using the Equation (1):

% Recovery_standards_ = standards _pretreated by the LC-18 SPE tube_/standards _without pretreatment_ × 100 (1)

The presence of organic acid interfering substances was verified by sample recovery rate. Three hawthorn wine samples were diluted five-fold with purified water after centrifugation; and each sample was aliquoted into two groups with the same treatment as organic acid standards. The calculation was performed using the Equation (2):

% Recovery_samples_ = samples _pretreated by the LC-18 SPE tube_/samples _without pretreatment_ × 100 (2)

In order to validate the proposed method, a fortified recovery test was conducted by 5 mL of analyzing samples fortified with 5 mL of certain concentration of the mixed standards solution. The calculation was carried out using the Equation (3):

% Recovery = 2 × analyte _mixture of standards and samples_/(analyte_sample_+ analyte_standard_) × 100 (3)

### 3.8. Statistical Analysis

Data were indicated as means ± SD of three replicates. Statistical analysis was performed using SPSS software version 22.0 (SPSS-IBM Inc, Chicago, IL, USA). One-way analysis of variance (ANOVA) was applied to analyze the difference of the results from HPLC method 1, HPLC method 2 and enzymatic method. Mean differences at *p* < 0.05 were considered to be significant using Tukey test. Sigmaplot 12.0 was used to draw HPLC chromatograms.

## 4. Conclusions

Two HPLC methods differed greatly in the degree of separation, analytical time and accuracy in determination organic acids in the hawthorn wines. A clean-up pretreatment of LC-18 SPE tube removed the impurity effectively, resulting in a significantly shortened elution period when the sample detected by an 87H column with a DAD detector at 215 nm (HPLC method 1). Citric and lactic acids were quantified accurately with this method. Using a dC18 column equipped with DAD detector at 210 nm (HPLC method 2), citric, succinic, l-malic, acetic, pyruvic, and fumaric acids were quantified reliably with the results of enzymatic method as references, even without the pretreatment of LC-18 SPE tube. It was concluded that HPLC method 2 was an effective method to quantify the organic acids in hawthorn wine. To our knowledge, it was the first time these two HPLC methods were compared for the quantification of organic acids in hawthorn wine. All organic acids analyzed in this study were taking enzymatic method as a reference, making the measurement results more accurate and reliable. In addition, this study also provided a method for quantifying the main organic acids simultaneously and accurately, and a good choice for quantification organic acids in other drinks could be suggested.

## Figures and Tables

**Table 1:** Physicochemical indices of hawthorn wine.

Samples	Alcohol (%, *v*/*v*)	Reducing Sugar (g/L)	Sugar-Free Extract (g/L)	Total Acidity (g/L)	pH
1	14.69 ± 0.01 ^b^	5.60 ± 0.04 ^a^	37.12 ± 0.06 ^a^	14.51 ± 0.00 ^b,c^	3.22 ± 0.01 ^c^
2	14.83 ± 0.01 ^b^	5.53 ± 0.07 ^a^	36.77 ± 0.38 ^a,b^	13.99 ± 0.00 ^c,d^	3.21 ± 0.00 ^c^
3	15.39 ± 0.01 ^a^	4.28 ± 0.00 ^c^	32.35 ± 0.18 ^e^	13.15 ± 0.09 ^e^	3.28 ± 0.01 ^a^
4	15.34 ± 0.00 ^a^	4.00 ± 0.07 ^c^	35.13 ± 0.33 ^c,d^	14.19 ± 0.04 ^c^	3.08 ± 0.00 ^f^
5	14.89 ± 0.01 ^b^	5.10 ± 0.07 ^b^	34.78 ± 0.03 ^d^	13.51 ± 0.02 ^d,e^	3.25 ± 0.00 ^b^
6	15.42 ± 0.20 ^a^	4.11 ± 0.09 ^c^	37.19 ± 0.47 ^a^	15.16 ± 0.04 ^a^	3.25 ± 0.00 ^b^
7	14.95 ± 0.01 ^b^	5.15 ± 0.07 ^b^	36.36 ± 0.01 ^a,b,c^	14.97 ± 0.00 ^a,b^	3.09 ± 0.00 ^e,f^
8	15.45 ± 0.07 ^a^	5.75 ± 0.11 ^a^	35.56 ± 0.62 ^b,c,d^	15.07 ± 0.05 ^a^	3.14 ± 0.01 ^d^
9	14.95 ± 0.01 ^b^	3.51 ± 0.12 ^d^	36.83 ± 0.35 ^a,b^	13.40 ± 0.11 ^e^	3.28 ± 0.01 ^a^
10	14.84 ± 0.00 ^b^	5.18 ± 0.04 ^b^	37.45 ± 0.30 ^a^	15.14 ± 0.03 ^a^	3.10 ± 0.00 ^e^

All values are expressed as means ± standard deviation (*n* = 3); different lowercase letters on the right in the same column indicated significant difference at the 95% confidence level (*p* < 0.05).

**Table 2:** Identification of organic acids with enzymatic method.

Samples	Methods	Citric Acid (mg/L)	Succinic Acid (mg/L)	l-malic Acid (mg/L)	Acetic Acid (mg/L)	Lactic Acid (mg/L)	Pyruvic Acid (mg/L)	Fumaric Acid (mg/L)	TOA (g/L)
1	HPLC 1 ^a^	9587.5 ± 150.3 ^a^	2830.1 ± 62.2 ^a^	772.1 ± 50.2 ^a^	993.8 ± 158.2 ^a^	88.9 ± 6.2 ^b^	1.4 ± 0.2 ^b^	1.1 ± 0.1 ^c^	14.3 ± 0.4
	HPLC 2 ^b^	9928.7 ± 274.2 ^a^	1611.2 ± 91.8 ^b^	835.4 ± 1.7 ^a^	740.5 ± 10.0 ^a^	940.2 ± 6.2 ^a^	7.0 ± 0.1 ^a^	2.5 ± 0.1 ^b^	14.1 ± 0.4
	EM ^c^	9372.1 ± 214.2 ^a^	1522.8 ± 47.2 ^b^	812.3 ± 2.8 ^a^	679.4 ± 7.2 ^a^	95.2 ± 2.3 ^c^	7.4 ± 0.3 ^a^	3.4 ± 0.6 ^a^	12.5 ± 0.3
2	HPLC 1	9315.4 ± 133.0 ^a^^,^^b^	2919.8 ± 1.8 ^a^	734.4 ± 7.5 ^a^	1073.9 ± 2.1 ^a^	86.5 ± 9.0 ^b^	0.9 ± 0.0 ^b^	1.1 ± 0.0 ^c^	14.1 ± 0.2
	HPLC 2	9785.0 ± 246.7 ^a^	1771.9 ± 61.2 ^b^	762.7 ± 9.8 ^a^	736.5 ± 18.4 ^b^	910.7 ± 12.1 ^a^	6.0 ± 0.2 ^a^	2.8 ± 0.1 ^b^	14.0 ± 0.3
	EM	8896.9 ± 192.0 ^b^	1571.6 ± 29.0 ^c^	745.0 ± 21.3 ^a^	643.9 ± 17.9 ^c^	103.0 ± 1.1 ^b^	6.2 ± 0.5 ^a^	3.7 ± 0.0 ^a^	12.0 ± 0.3
3	HPLC 1	8864.9 ± 282.9 ^a^	3110.3 ± 155.3 ^a^	484.1 ± 123.9 ^a^	892.1 ± 150.6 ^a^	102.3 ± 26.4 ^b^	1.3 ± 0.1 ^c^	1.2 ± 0.1 ^c^	13.5 ± 0.7
	HPLC 2	8744.5 ± 62.0 ^b^	1797.1 ± 54.7 ^b^	591.6 ± 2.9 ^a^	662.3 ± 12.9 ^a^	1114.2 ± 13.0 ^a^	7.0 ± 0.2 ^a^	2.6 ± 0.0 ^b^	12.9 ± 0.1
	EM	7967.6 ± 88.7 ^c^	1694.9 ± 14.5 ^b^	603.2 ± 14.2 ^a^	598.3 ± 10.8 ^a^	120.8 ± 2.7 ^c^	5.6 ± 0.5 ^b^	3.3 ± 0.0 ^a^	11.0 ± 0.1
4	HPLC 1	10230.0 ± 257.4 ^a^	2993.0 ± 23.1 ^a^	782.5 ± 33.4 ^b^	720.6 ± 56.4 ^a^	94.6 ± 9.5 ^b^	2.6 ± 0.0 ^c^	1.6 ± 0.2 ^b^	14.8 ± 0.4
	HPLC 2	9104.4 ± 80.8 ^b^	1753.8 ± 28.8 ^b^	681.5 ± 1.5 ^c^	665.2 ± 5.0 ^b^	1127.6 ± 5.1 ^a^	8.9 ± 0.4 ^a^	2.5 ± 0.0 ^a^	13.3 ± 0.1
	EM	9821.3 ± 132.6 ^a^	1664.1 ± 14.5 ^c^	983.2 ± 31.3 ^a^	694.6 ± 7.2 ^a^	106.1 ± 1.8 ^c^	4.9 ± 0.7 ^b^	2.5 ± 0.1 ^a^	13.3 ± 0.2
5	HPLC 1	9242.9 ± 130.8 ^a^	3092.1 ± 54.8 ^a^	742.3 ± 62.5 ^a^	959.1 ± 163.5 ^a^	87.6 ± 14.^6 b^	1.1 ± 0.1 ^b^	1.1 ± 0.1 ^c^	14.1 ± 0.4
	HPLC 2	9196.6 ± 4.4 ^a^	1741.6 ± 30.3 ^b^	716.4 ± 6.2 ^a^	694.3 ± 3.3 ^a^	950.7 ± 5.3 ^a^	6.5 ± 0.2 ^a^	2.8 ± 0.1 ^b^	13.3 ± 0.0
	EM	8881.3 ± 95.9 ^a^	1640.9 ± 32.7 ^b^	716.8 ± 1.4 ^a^	616.0 ± 21.5 ^a^	89.9 ± 1.6 ^c^	6.9 ± 1.3 ^a^	3.3 ± 0.3 ^a^	12.0 ± 0.2
6	HPLC 1	9493.9 ± 6.1 ^a^	3261.9 ± 5.0 ^a^	501.7 ± 54.8 ^a^	982.6 ± 24.1 ^a^	86.9 ± 14.6 ^b^	2.0 ± 0.1 ^c^	1.7 ± 0.0 ^c^	14.3 ± 0.1
	HPLC 2	9321.1 ± 286.4 ^a^	1668.6 ± 111.3 ^b^	733.1 ± 5.2 ^a^	683.2 ± 2.0 ^b^	1131.2 ± 3.4 ^a^	7.5 ± 0.2 ^a^	2.7 ± 0.1 ^b^	13.5 ± 0.4
	EM	8855.2 ± 0.0 ^a^	1607.6 ± 58.1 ^b^	701.7 ± 93.8 ^a^	603.3 ± 17.9 ^c^	102.4 ± 1.6 ^c^	4.8 ± 0.1 ^b^	3.5 ± 0.2 ^a^	11.9 ± 0.2
7	HPLC 1	9111.0 ± 472.2 ^a^	2613.4 ± 156.3 ^a^	771.5 ± 11.3 ^a^	866.1 ± 162.6 ^a^	66.0 ± 6.1 ^b^	0.9 ± 0.3 ^c^	1.0 ± 0.2 ^c^	13.4 ± 0.8
	HPLC 2	9709.1 ± 158.4 ^a^	1573.4 ± 36.8 ^b^	877.7 ± 2.8 ^a,b^	761.1 ± 15.6 ^a^	795.0 ± 2.6 ^a^	7.1 ± 0.3 ^a^	2.4 ± 0.1 ^b^	13.7 ± 0.2
	EM	9090.0 ± 0.0 ^a^	1468.9 ± 14.5 ^b^	878.7 ± 42.6 ^b^	723.7 ± 19.7 ^a^	86.2 ± 6.8 ^c^	5.3 ± 0.7 ^b^	2.8 ± 0.0 ^a^	12.3 ± 0.1
8	HPLC 1	9753.4 ± 140.8 ^b^	2165.8 ± 55.9 ^a^	701.3 ± 20.2 ^a^	1111.9 ± 127.9 ^a^	61.9 ± 0.7 ^b^	0.6 ± 0.1 ^b^	1.0 ± 0.1 ^c^	13.8 ± 0.3
	HPLC 2	10156.6 ± 113.3 ^a^	1456.0 ± 30.6 ^b^	756.3 ± 0.3 ^a^	908.1 ± 17.7 ^b^	740.1 ± 3.8 ^a^	5.9 ± 0.2 ^a^	2.5 ± 0.0 ^b^	14.0 ± 0.2
	EM	9173.7 ± 44.3 ^c^	1327.7 ± 3.6 ^b^	754.0 ± 37.0 ^a^	818.8 ± 0.0 ^b^	72.7 ± 0.5 ^c^	6.1 ± 0.4 ^a^	3.3 ± 0.4 ^a^	12.2 ± 0.1
9	HPLC 1	9069.4 ± 61.5 ^a^	3141.3 ± 66.8 ^a^	576.5 ± 137.1 ^a^	760.8 ± 106.7 ^a^	105.9 ± 14.4 ^b^	2.5 ± 0.2 ^c^	1.3 ± 0.1 ^c^	13.7 ± 0.4
	HPLC 2	8843.8 ± 61.9 ^a^	1857.4 ± 47.6 ^b^	659.2 ± 0.1 ^a^	616.8 ± 7.8 ^b^	1140.2 ± 1.9 ^a^	8.8 ± 0.0 ^a^	2.8 ± 0.0 ^b^	13.1 ± 0.1
	EM	8145.1 ± 103.4 ^b^	1540.8 ± 21.8 ^c^	670.6 ± 44.1 ^a^	523.5 ± 1.8 ^a,b^	135.4 ± 2.5 ^c^	5.0 ± 0.4 ^b^	3.6 ± 0.3 ^a^	11.0 ± 0.2
10	HPLC 1	9570.5 ± 221.0 ^a^	2748.1 ± 68.6 ^a^	798.0 ± 16.0 ^b^	1252.1 ± 62.8 ^a^	140.6 ± 14.9 ^b^	3.9 ± 0.3 ^c^	1.2 ± 0.2 ^c^	14.5 ± 0.4
	HPLC 2	10473.9 ± 454.4 ^a^	1655.4 ± 82.2 ^b^	956.3 ± 16.7 ^a^	734.6 ± 2.3 ^c^	881.0 ± 12.2 ^a^	7.7 ± 0.0 ^a^	2.6 ± 0.0 ^b^	14.7 ± 0.6
	EM	9460.9 ± 169.8 ^a^	1499.7 ± 21.8 ^b^	911.9 ± 35.5 ^a^	694.6 ± 10.8 ^b^	80.7 ± 0.0 ^c^	5.6 ± 0.5 ^b^	3.2 ± 0.9 ^a^	12.7 ± 0.2

^a^ HPLC 1, by an 87H column equipped with DAD detector at 215 nm (HPLC method 1); ^b^ HPLC 2, by a dC18 column equipped with DAD detector at 210 nm (HPLC method 2). ^c^ EM, enzymatic method; TOA, total organic acid. Data are expressed as mean ± standard deviation. Different lowercase letters in the same column of same sample are significantly different by a Tukey test (*p* < 0.05).

**Table 3:** Validation parameters for the two HPLC methods.

Methods	OA	Concentration (mg/L)	Equation	R^2^	Precision%	R%	LOD (mg/L)	LOQ (mg/L)
HPLC 1	Citric	606.6–12411.00	Y = 1879.80x + 8077.20	1.0000	0.76	100.4	0.61	2.02
	Pyruvic	0.00–8.90	Y = 31419.00x + 1474.70	0.9995	4.21	92.2	0.03	0.11
	l-malic	72.11–1730.69	Y = 1731.50x + 1210.30	0.9999	1.72	101.2	0.64	1.60
	Succinic	128.88–1611.00	Y = 945.26x − 1668.30	0.9999	0.76	99.4	1.75	5.84
	Lactic	1.68–84.00	Y = 815.37x − 629.03	1.0000	3.66	91.1	1.93	6.45
	Fumaric	0.09–4.7	Y = 199715x − 5007.3	0.9999	2.95	94.4	0.01	0.03
	Acetic	1.68–427.20	Y = 802.29x − 355.28	1.0000	0.88	83.6	2.11	7.05
	Citric	426.24–10656.00	Y = 1011.90x − 15648.00	1.0000	0.57	100.9	1.16	3.86
HPLC 2	Pyruvic	0.00–30.20	Y = 13348.00x − 732.84	0.9999	1.28	100.3	0.08	0.28
	l-malic	56.10–1402.4	Y = 797.75x + 2302.9	0.9999	0.48	102.8	1.00	3.33
	Succinic	122.48–3062.00	Y = 605.52x − 6762.2	0.9999	1.73	100.1	2.13	7.10
	Lactic	2.45–522.0	Y = 475.82x + 1562.20	0.9998	0.26	100.7	1.04	3.46
	Fumaric	0.00–7.36	Y = 134887.00x + 4235.6	0.9999	0.53	98.2	0.01	0.03
	Acetic	10.89–544.40	Y = 540.39x + 476.03	0.9999	0.54	108.5	1.98	6.6

OA: organic acid; R%: recovery; LOD: limit of detection; LOQ: limit of quantification.

**Table 4:** Recovery of organic acids treated with LC-18 SPE tube.

Method	Organic Acid	Retention Time (min)	Precision %	LOD	LOQ	Recovery in Standards (%)	Recovery in Samples (%)	Fortified Recovery (%)
HPLC 1	Citric	7.5	2.22	0.65	2.18	93.2 ± 1.8	101.9 ± 3.7	103.6 ± 1.9
	Pyruvic	8.5	5.01	0.03	0.11	97.7 ± 1.0	139.9 ± 34.9	120.7 ± 8.8
	l-malic	9.2	3.68	0.69	2.29	93.2 ± 1.8	107.0 ± 8.4	115.6 ± 2.0
	Succinic	11.2	2.59	1.73	6.42	90.8 ± 6.7	93.9 ± 4.7	94.7 ± 1.3
	Lactic	12.1	4.32	1.90	6.33	101.6 ± 4.2	71.4 ± 16.3	70.4 ± 3.5
	Fumaric	13.5	4.12	0.01	0.04	94.0 ± 2.6	92.5 ± 3.4	94.49 ± 0.3
	Acetic	14.5	4.48	2.14	7.13	98.6 ± 2.6	88.7 ± 16.6	81.8 ± 5.6
HPLC 2	Citric	9.9	2.36	1.17	3.90	99.2 ± 2.3	99.0 ± 1.4	100.9 ± 2.1
	Pyruvic	6.1	3.23	0.08	0.28	94.0 ± 9.2	99.7 ± 4.0	100.3 ± 0.7
	l-malic	6.4	0.61	1.00	3.33	100.8 ± 0.1	100.2 ± 2.9	102.8 ± 0.1
	Succinic	10.9	5.62	2.21	7.38	96.2 ± 5.4	95.1 ± 1.8	100.1 ± 2.7
	Lactic	7.4	0.70	1.03	3.42	101.3 ± 3.2	100.6 ± 3.1	100.7 ± 0.8
	Fumaric	12.4	3.28	0.01	0.03	91.2 ± 8.1	94.7 ± 2.8	98.2 ± 7.3
	Acetic	7.8	2.06	1.92	6.40	103.1 ± 4.4	101.7 ± 9.4	108.5 ± 10.1

LOD: limit of detection; LOQ: limit of quantification. Results are expressed as mean ± SD (standard deviation) (*n* = 3).

**Table 5:** Organic acids in hawthorn fruits.

Organic Acid	Citric	Succinic	l-malic	Acetic	Lactic	Pyruvic	Fumaric	TOA
Content (mg/g)	26.95 ± 0.41	0.63 ± 0.05	3.29 ± 0.08	0.75 ± 0.13	0.06 ± 0.00	0.02 ± 0.00	nd	31.70 ± 0.67

nd, below detectable limit; all values are expressed as means ± standard deviation (*n* = 3).

## References

[1] Rigelsky J.M., Sweet B.V. (2002). Hawthorn wine: Pharmacology and therapeutic uses. Am. J. Health Syst. Ph..

[2] Zhang Z.S., Ho W.K.K., Huang Y., Chen Z.Y. (2002). Hypocholesterolemic activity of hawthorn wine fruit is mediated by regulation of cholesterol-7alpha-hydroxylase and acyl COA: Cholesterol acyltransferase. Food Res. Int..

[3] Quettier-Deleu C., Voiselle G., Fruchart J.C., Duriez P., Teissier E., Bailleul F., Vasseur J., Trotin F. (2003). Hawthorn wine extracts inhibit LDL oxidation. Pharmazie.

[4] Tassell M.C., Rosari K., Deirdre G., Mary L., Ambrose F. (2010). Hawthorn wine (Crataegus spp.) in the treatment of cardiovascular disease. Pharm. Rev..

[5] Zhu R.G., Sun Y.D., Li T.P., Chen G., Peng X., Duan W.B., Zheng Z.Z., Shi S.L., Xu J.G., Liu Y.H. (2015). Comparative effects of hawthorn wine (Crataegus pinnatifida, bunge) pectin and pectin hydrolyzates on the cholesterol homeostasis of hamsters fed high-cholesterol diets. Chem. Biol. Interact..

[6] Aierken A., Buchholz T., Chen C., Zhang X., Melzig M.F. (2017). Hypoglycemic effect of hawthorn wine in type II diabetes mellitus rat model. J. Sci. Food Agr..

[7] Liu P.Z., Kallio H., Lü D.G., Zhou C.S., Ou S.Y., Yang B.R. (2010). Acids, sugars, and sugar alcohols in Chinese hawthorn wine (Crataegus spp.) fruits. J. Agr. Food Chem..

[8] Silva F.L.D.N., Schmidt E.M., Messias C.L., Eberlin M.N. (2014). Quantitation of organic acids in wine and grapes by direct infusion electrospray ionization mass spectrometry. Anal. Methods UK.

[9] Hu J.T., Du J.H., He G.F. (2012). Influence of fruit yeasts on quality and antioxidant capability of hawthorn wine wine. Liquor making.

[10] He G.F., Sui J.L., Du J.H., Lin J. (2013). Characteristics and antioxidant capacities of five hawthorn wine wines fermented by different wine yeasts. J. Inst. Brewing.

[11] Liu S.W., Zhang X., You L., Guo Z.Y., Chang X.D. (2018). Changes in anthocyanin profile, color, and antioxidant capacity of hawthorn wine wine (Crataegus pinnatifida) during storage by pretreatments. LWT Food Sci. Technol..

[12] Liu S.W., Chang X.D., Liu X.F., Shen Z.E. (2016). Effects of pretreatments on anthocyanin composition, phenolics contents and antioxidant capacities during fermentation of hawthorn wine (Crataegus pinnatifida) drink. Food Chem..

[13] Dong W.J., Du J.H., Fu Y.Z. (2015). Research of hawthorn wine wine with reduced methanol content. Liquor Making.

[14] Anthon G.E., Lestrange M., Barrett D.M. (2011). Changes in pH, acids, sugars and other quality parameters during extended vine holding of ripe processing tomatoes. J. Sci. Food Agr..

[15] Velterop J.S., Vos F. (2001). A rapid and inexpensive microplate assay for the enzymatic determination of glucose, fructose, sucrose, l-malate and citrate in tomato (Lycopersicon esculentum) extracts and in orange juice. Phytochem. Anal..

[16] Vermeir S., Nicolai B., Jans K., Maes G., Lammertyn J. (2007). High-throughput microplate enzymatic assays for fast sugar and acid quantification in apple and tomato. J. Agr. Food Chem..

[17] Arraezroman D., Fernandezsanchez J.F., Cortaceroramirez S., Seguracarretero A., Fernandezgutierrez A. (2006). A simple light-emitted diode-induced fluorescence detector using optical fibers and a charged coupled device for direct and indirect capillary electrophoresis methods. Electrophoresis.

[18] Peres R.G., Moraes E.P., Micke G.A., Tonin F.G., Tavares M.F.M., Rodriguez-Amaya D.B. (2009). Rapid method for the determination of organic acids in wine by capillary electrophoresis with indirect UV detection. Food Control..

[19] Jham G.N., Fernandes S.A., Garcia C.F., Da S.A. (2002). Comparison of GC and HPLC for the quantification of organic acids in coffee. Phytochem. Anal..

[20] Stelios K., Paraskeri S., Theophiles M. (2007). Comparison of the characteristics of set type yoghurt made from ovine milk of different fat content. Int. J. Food Sci. Tech..

[21] Tanaka K., Mori M., Xu Q., Helaleh M.I.H., Ikedo M., Taoda H., Hu W., Hasebe K., Fritz J.S., Haddad P.R. (2003). Ion-exclusion chromatography with conductimetric detection of aliphatic carboxylic acids on a weakly acidic cation-exchange resin by elution with benzoic acid-β-cyclodextrin. J. Chromatogr. A..

[22] Tormo M., Izco J.M. (2004). Alternative reversed-phase high-performance liquid chromatography method to analyse organic acids in dairy products. J. Chromatogr. A..

[23] Scherer R., Rybka A.C.P., Ballus C.A., Meinhart A.D., Filho J.T., Godoy H.T. (2012). Validation of a HPLC method for simultaneous determination of main organic acids in fruits and juices. Food Chem..

[24] Coelho E.M., Padilha C.V., Miskinis G.A., De Sá A.G.B., Pereira G.E., Azevedo L., Lima M.D. (2018). Simultaneous analysis of sugars and organic acids in wine and grape juices by HPLC: Method validation and characterization of products from Northeast Brazil. J. Food Compos. Anal..

[25] Pereira V., Câmara J.S., Cacho J., Marques J.C. (2010). HPLC-DAD methodology for the quantification of organic acids, furans and polyphenols by direct injection of wine samples. J. Sep. Sci..

[26] Kadakal C., Nas S. (2002). Effect of activated charcoal on patulin, fumaric acid and some other properties of apple juice. Die Nahrung.

[27] Gökmen V., Acar J. (2004). Fumaric acid in apple juice: A potential indicator of microbial spoilage of apples used as raw material. Food Addit Contam..

[28] Shapiro F., Silanikove N. (2011). Rapid and accurate determination of malate, citrate, pyruvate and oxaloacetate by enzymatic reactions coupled to formation of a fluorochromophore: Application in colorful juices and fermentable food (yogurt, wine) analysis. Food Chem..

[29] Konieczka P. (2007). The role of and the place of method validation in the quality assurance and quality control (QA/QC) system. Crit. Rev. Anal. Chem..

[30] Gao Y., Ierapetritou M.G., Muzzio F.J. (2013). Determination of the confidence interval of the relative standard deviation using convolution. J. Pharm. Innov..

[31] Agius C., Tucher S.V., Poppenberger B., Rozhon W. (2018). Quantification of sugars and organic acids in tomato fruits. Methodsx.

[32] Gałuszka A., Migaszewski Z.M., Konieczka P., Namieśnik J. (2012). Analytical Eco-Scale for assessing the greenness of analytical procedures. Trends Anal. Chem..

[33] Płotka-wasylka J. (2018). A new tool for the evaluation of the analytical procedure: Green analytical procedure index. Talanta.

[34] Cristina G.M., Luh B.S. (2010). HPLC analysis of organic acids and sugars in tomato juice. J. Food Sci..

[35] Marconi O., Floridi S., Montanari L. (2007). Organic acids profile in tomato juice by HPLC with UV detection. J. Food Qual..

[36] Rozhon W., Petutschnig E., Wrzaczek M., Jonak C. (2005). Quantification of free and total salicylic acid in plants by solid-phase extraction and isocratic high-performance anion-exchange chromatography. Anal. Bioanal. Chem..

